# Antiviral Activity of Medicinal Plant Extracts *Vitex negundo* and *Macaranga tanarius* Against SARS-CoV-2

**DOI:** 10.3390/pathogens14080820

**Published:** 2025-08-19

**Authors:** Muhareva Raekiansyah, Mya Myat Ngwe Tun, Alexandra Ang, Alexandra Lee, Stephani Joy Macalino, Junie Billones, Yuki Takamatsu, Takeshi Urano, Lyre Anni E. Murao, Noel Quiming, Kouichi Morita, Maria Constancia Carrillo

**Affiliations:** 1 Department of Tropical Viral Vaccine Development, Institute of Tropical Medicine, Nagasaki University, Nagasaki 852-8523, Japan; m.raekiansyah@nagasaki-u.ac.jp; 2 Department of Virology, Institute of Tropical Medicine, Nagasaki University, Nagasaki 852-8523, Japan; yukiti@nagasaki-u.ac.jp; 3 Center for Vaccines and Therapeutic Antibodies for Emerging Infectious Diseases, Shimane University, Izumo 690-8504, Japan; turano@med.shimane-u.ac.jp; 4 DEJIMA Infectious Diseases Research Alliance, Nagasaki University, Nagasaki 852-8523, Japan; 5 Department of Physical Sciences and Mathematics, College of Arts and Sciences, University of the Philippines Manila, Manila 1000, Philippines; adang3@up.edu.ph (A.A.); aplee2@up.edu.ph (A.L.); jbbillones@up.edu.ph (J.B.); nsquiming@up.edu.ph (N.Q.); mtobrerocarrillo@up.edu.ph (M.C.C.); 6 Department of Chemistry, College of Science, De La Salle University, Manila 0922, Philippines; stephani.macalino@dlsu.edu.ph; 7 Department of Biological Science and Environmental Studies, College of Science and Mathematics, University of the Philippines Mindanao, Davao 8000, Philippines; lemurao@up.edu.ph

**Keywords:** SARS-CoV-2, antiviral, *Vitex negundo*, *Macaranga tanarius*

## Abstract

Natural products possess a wide range of biological and biochemical potentials, with plant-derived compounds being significant sources for discovering new drugs. In this study, extracts of *Vitex negundo* and *Macaranga tanarius* prepared with different solvents were tested for their antiviral activity against the original SARS-CoV-2 Wuhan strain and its variants using plaque assay, quantitative real time RT-PCR, and immunofluorescence assay (IFA). Our results showed that at their maximum non-toxic concentrations, Vitex-Dichloromethane (DCM) and Macaranga extracts significantly inhibited SARS-CoV-2 Wuhan strain growth in Vero E6 cells, showing a 5-log reduction in plaque assay and confirmed by IFA. Meanwhile, Vitex-Hexane showed moderate activity with a 2-log decrease. The inhibition was shown in a dose-dependent manner. The antiviral efficacy of these extracts was further demonstrated against various SARS-CoV-2 variants including Alpha, Beta, Delta, and Omicron. Both Vitex-DCM and Macaranga showed strong virucidal activity. In addition, Vitex-DCM and Macaranga inhibited the transcriptional activity of purified SARS-CoV-2 RdRp, indicating that RdRp inhibition may contribute to viral suppression as shown at the post-infection stage. Furthermore, combining Vitex-DCM or Macaranga with remdesivir showed a synergistic effect against SARS-CoV-2. These results suggest that *Vitex negundo* and *Macaranga tanarius* extracts are promising candidates for anti-SARS-CoV-2 treatments. Their synergy with remdesivir also underscores the potential of drug combinations in fighting SARS-CoV-2 and preventing the emergence of mutant variants.

## 1. Introduction

Human coronaviruses (HCoVs) continue to pose significant global threats to public health, having been responsible for several outbreaks and pandemics throughout history. The first major outbreak was caused by the severe acute respiratory syndrome coronavirus (SARS-CoV), which emerged in 2002–2003 [1]. In December 2019, another novel coronavirus, later named severe acute respiratory syndrome coronavirus 2 (SARS-CoV-2), was identified as the causative agent of coronavirus disease 2019 (COVID-19). This outbreak began in Wuhan City, Hubei Province, China [2]. SARS-CoV-2 triggered a global pandemic, resulting in millions of deaths and causing severe economic and societal disruptions worldwide [3].

Following the emergence of the SARS-CoV-2 pandemic, identifying effective treatments has become a priority. Several vaccines have been developed and approved in response to the COVID-19 pandemic. These vaccines have been administered to millions of people worldwide and have been shown to reduce severe disease and mortality, as well as lower the risk of SARS-CoV-2 reinfection and hospitalization [4,5]. In addition, antiviral drug development for SARS-CoV-2 has been pursued through various approaches, including drug repurposing.

Several potential treatments against SARS-CoV-2 have been proposed, including neuraminidase inhibitors [6], remdesivir [7], and protease inhibitors [8]. However, the continuous emergence of new SARS-CoV-2 variants due to virus evolution highlights the need for the development of additional therapeutic strategies alongside existing treatments, including those derived from plants.

Plants or herbal products are widely recognized as a valuable source of bioactive molecules for the discovery and development of new antiviral drugs. They are ideal candidates for new drugs due to their chemical variety, limited substantial toxic effects, abundant availability in nature, and broad-spectrum antiviral properties [9,10]. The effectiveness of medicinal plants correlates with the presence of specific active substances.

An increasing number of studies have investigated the antiviral activities of plant-derived compounds and extracts against SARS-CoV-2 [11]. In addition to their antiviral activity, the plant products have also been shown to exhibit immunomodulatory effects by regulating the excessive release of cytokines, a phenomenon often linked to SARS-CoV-2 infections [12]. During the COVID-19 pandemic, traditional herbal medicines have been utilized alongside conventional treatments to help in the management of the disease. These herbal remedies have been shown to inhibit SARS-CoV-2 replication or associated with a reduced progression rate from mild or moderate cases to severe cases [13,14,15].

The Philippines is known for its rich biodiversity, yet many plant species have not been extensively explored for their potential as antivirals. Some Philippine medicinal plants with potential anti-SARS-CoV-2 activities have been suggested [16,17]. For example, *Vitex negundo* L. (Verbenaceae), commonly known as *lagundi*, was tested in a clinical trial during the pandemic as an adjunctive treatment for COVID-19 [18]. Indeed, *Vitex negundo* has long been used as a folk medicine in the Philippines and other Asian countries. This ethnobotanically important plant possesses numerous medicinal properties, including antimalarial [19], anti-inflammatory, anticancer, and antimicrobial effects [20]. Another medicinal plant that grows throughout eastern and southern Asia, including the Philippines, is *Macaranga tanarius*. Macaranga has long been used in folk medicine, and its extracts have demonstrated various therapeutic potentials, including antibacterial and antifungal properties [21] as well as free radical scavenging activity [22]. Although many other medicinal plants from the Philippines are believed to have antiviral activity, including against SARS-CoV-2, there is still a lack of studies that investigate and validate their potential. A few studies have employed in-silico screening methods for this purpose [23,24]; however, in vitro or in vivo studies remain very limited. Therefore, in this study, we investigated several medicinal plant extracts from the Philippines for their potential as sources for the development of anti-SARS-CoV-2 drugs.

## 2. Materials and Methods

### 2.1. Sample Preparation and Extraction

Leaves of *Macaranga tanarius* were collected from the Hijo Estate Resorts Forest, Brgy. Madaum, Tagum City, Davao del Norte. The leaves were lyophilized for 5 days or until completely dry. Dried biomass was then ground into powder, and fifty grams of the powdered material was subjected to a 48 h maceration in 95% ethanol. Maceration was performed thrice for each jar to ensure maximum yield. The extract was collected by vacuum filtration and the solvent was removed by rotary evaporation. Crude extracts were then transferred to conical tubes and stored at −40 °C prior to use. The crude extracts were prepared for in vitro assays by reconstituting in dimethylsulfoxide (DMSO). Vitex negundo seeds were generously supplied by the National Integrated Research Program on Medicinal Plants-Institute of Herbal Medicine (NIRPROMP-IHM) from their plantation in Los Banos, Laguna, Philippines. A total of 93.5 g of the seeds was harvested and air-dried prior to maceration in 80% ethanol and shaking for 2 h. Filtration was performed three times through Whatman filter paper, with the residue re-macerated before every filtration. The final pool filtrate was then subjected to rotary evaporation to remove the ethanol, resulting in a water-rich crude extract. Liquid–liquid extraction with equal volumes of the water-rich extract to solvent was done successively first with hexane, then dichloromethane (DCM), then ethyl acetate (EtOAc), and lastly with n-butanol (n-BuOH). The residues collected were (1) 0.1029 g from the hexane fraction, (2) 0.1046 g from the DCM fraction, (3) 0.1917 g from the EtOAc fraction, and (4) 0.0116 g from the n-BuOH fraction. These fractions were transported to Nagasaki University, Japan for the in vitro assays.

### 2.2. Virus and Cells

SARS-CoV-2 (Wuhan strain) and its variants (Alpha, Beta, Delta, and Omicron) provided by the Japan National Institute of Infectious Diseases were propagated in Vero-E6 cells (African green monkey kidney cells) and cultured in minimum essential medium (MEM) supplemented with 10% fetal calf serum (FCS). Five days post-infection, the supernatant was collected, centrifuged at 2500 rpm for 10 min, and stored at −80 °C as virus stock. For virus quantification by plaque assay, Vero TM was used with the same media. All experiments involving infectious SARS-CoV-2 were conducted in a biosafety level 3 (BSL-3) laboratory at Nagasaki University, adhering to standard BSL-3 safety protocols.

### 2.3. Cell Viability Assay

The viability of Vero E6 cells after exposure the samples extract was evaluated using 3-(4,5-dimethylthiazol-2-yl)-2,5-diphenyl tetrazolium bromide (MTT) according to the manufacturer’s instructions (Promega, Madison, WI, USA). In brief, confluent Vero cells E6 in 96-well plates were treated with different concentrations of samples. After 48 h, 30 µL/well of MTT (at final concentration of 0.2 mg/mL) was added to each well and the plates were incubated for 4 h at 37 °C, with 5% CO_2_. Finally, 100 µL/well of Solubilization/Stop Solution was added and incubated overnight. The optical density was measured at 570 nm using a microplate reader (Synergy H1 M, Biotech, USA). Cell viability was calculated as follows: cell viability (%) = [(sample OD_570_)/(cell control OD_570_) × 100]. Concentrations with cell viability after treatment of 80% or more were considered non-cytotoxic.

### 2.4. Evaluation of the Antiviral Activity Against SARS-CoV-2

The antiviral activity of the extract samples was initially evaluated using a full or simultaneous treatment strategy (all stages of infection) as previously described [25]. Briefly, confluent Vero E6 cells in 96-well plates were infected with the SARS-CoV-2 Wuhan strain or the Alpha, Beta, Delta, or Omicron variants at a multiplicity of infection (MOI) of 0.05–0.1. The infections were conducted in the presence of the extract samples or remdesivir, which served as a positive control for inhibition, at the indicated concentrations. Untreated infected cells were used as a negative control. The cells were incubated for 48 h at 37 °C under a 5% CO_2_ atmosphere. After incubation, the infectious culture fluid (ICF) was harvested and subjected to a plaque assay for virus quantification. Inhibition of SARS-CoV-2 variants by the extract samples or remdesivir was evaluated by quantifying the virus from the ICF using real-time RT-PCR. In a separate experiment, the cells were fixed and stained for an immunofluorescence assay (IFA).

### 2.5. Time-of-Addition Assay

Time-of-addition studies were performed in 96-well plate cells as follows. (i) Pre-treatment of virus: SARS-CoV-2 Wuhan strain at an MOI of 0.05 was pre-incubated with the extract samples or remdesivir at the indicated concentrations on ice for 2 h. Virus mixed with media only was used as a negative control. The mixture was then added to Vero E6 cells and incubated at 37 °C for 2 h. After 1.5 h virus adsorption, the cells were subsequently washed twice with PBS and cultured in fresh medium for 48 h. (ii) During-infection assay: Vero E6 cells were infected with the SARS-CoV-2 Wuhan strain at a multiplicity of infection (MOI) of 0.05 in the presence of extract samples or remdesivir at the indicated concentrations. Infected cells without treatment were used as an infection control. After 1.5 h of viral adsorption, the inoculum was removed and the cells were washed with phosphate-buffered saline (PBS). The cells were then incubated in fresh medium for 48 h. (iii) After-infection assay: Vero cells were infected with the SARS-CoV-2 Wuhan strain at a multiplicity of infection (MOI) of 0.05. After 2 h viral adsorption, the cells were washed with phosphate-buffered saline and incubated in fresh medium containing extract samples or remdesivir at the indicated concentrations for 48 h. Infected cells without treatment were used as an infection control. For all experiments, culture fluids (ICFs) were harvested and subjected to a plaque assay and quantitative real-time RT-PCR for viral and RNA quantification. Additionally, the cells were fixed and stained for IFA.

### 2.6. Virus Titration and Quantification of Antiviral Activity by Plaque Assay

The virus titer and antiviral activity were determined by plaque assay in a 24-well plate. Briefly, tenfold serial dilutions of virus stock or infected cell supernatants (200 µL/well) were added to confluent Vero TM cells in duplicate. After 1.5 h of viral absorption, 0.5 mL of 1.25% methylcellulose 4000 (Wako, Osaka, Japan) in 2% FCS MEM was added and the plates were incubated at 37 °C with 5% CO_2_ for 5 days. For plaque staining, the media was removed and the cells were fixed overnight with 10% formaldehyde. The cells were then stained with a 1% crystal violet solution. Viral plaques were subsequently counted.

### 2.7. Immunofluorescence Assay (IFA)

Confluent Vero E6 cells in a 96-well plate were infected with SARS-CoV-2 in the presence of sample extracts. After 48 h, the media was removed and the cells were fixed with 4% paraformaldehyde (Wako, Osaka, Japan) for 1 h. Immunostaining was performed as previously described [26]. Briefly, after permeabilization of cells for 20 min at room temperature with 1% NP-40 lysis buffer (containing nonionic detergent NP-40 for membrane disruption) and blocking with BlockAce (Yukijirushi, Sapporo, Japan) at room temperature to minimize nonspecific binding, the cells were stained with a mouse antibody against the SARS-CoV-2 N protein as the primary antibody. For the secondary antibody, the cells were stained with Alexa Fluor 488–conjugated goat anti-mouse IgG (Invitrogen, Waltham, MA, USA). Finally, the cells were counterstained with DAPI (4′,6-Diamidino-2-phenylindole dihydrochloride) and the images were captured using a Keyence (BZ-X710) fluorescence microscope (Keyence, Itasca, IL, USA).

### 2.8. Quantification of Antiviral Activity by Quantitative Real Time RT-PCR (RT-qPCR)

A volume of 100 µL of infected cell supernatant was harvested and subjected to viral RNA extraction using the Nextractor NX-48 robot and NX-48S Viral NA Kit (Genolution Inc., Seoul, Republic of Korea) according to the manufacturer’s instructions. Five microliters of extracted RNA were used for one-step RT-qPCR to amplify the nucleocapsid (N) gene, as described in previous reports [26,27]. The 20 µL reaction mixture consisted of 5 µL of TaqMan master mix, 7 µL of nuclease-free water, 1 µL each of 0.5 µM forward and reverse primers, 0.25 µM probe, SARS-CoV-2 N-gene-specific primers, and TaqMan Fast Virus 1-Step Master Mix (Life Technologies, Carlsbad, CA, USA). Thermal cycling was performed on a QuantStudio 6 Pro real-time PCR system (Thermo Fisher Scientific, Waltham, MA, USA) with the following parameters: 50 °C for 5 min (reverse transcription) and 95 °C for 20 s (initial denaturation), followed by 40 cycles of 95 °C for 3 s and 60 °C for 30 s. The PCR runs were analyzed using QuantStudio^TM^ Real-Time PCR Software V1.7.2. An automatic threshold was applied in all assays to determine the threshold cycle (Ct). The number of SARS-CoV-2 RNA copies was determined by absolute quantification using viral RNA standards.

### 2.9. Inhibition Activity of SARS-CoV-2 RNA-Dependent RNA Polymerase (RdRp)

The in vitro activity of SARS-CoV-2 RdRp was assessed using the SARS-CoV-2 RNA-Dependent RNA Polymerase Kit Plus (Profoldin #S2RPA100KE, Hudson, MA, USA), following the manufacturer’s instructions. In brief, 50 µL reactions mixture which include purified RdRp proteins nsp7L8, nsp8, and nsp12 were incubated at 37 °C for 2 h in the presence or absence (control) of the extracts at indicated concentrations. The RdRp polymerization process was stopped by adding 130 µL of fluorescent dye solution. The resulting fluorescence intensity was measured at 485 nm excitation and 535 nm emission using microplate reader (Synergy H1 M, Biotech, Winooski, VT, USA). The transcriptional activity of control samples treated with 0.1% DMSO was considered 0% for calculating relative enzymatic inhibition, while 100% inhibition was determined using 10 µM remdesivir.

### 2.10. Evaluation of the Synergistic Effect of Vitex-DCM and Macaranga with Remdesivir

Vero E6 cells were seeded in a 96-well plate, and three dilutions of remdesivir (concentration: 0, 2.5, and 5 μM) and two dilutions of Vitex-DCM (concentration: 0 and 25 μg/mL) or Macaranga (0 and 3.12 μg/mL) were tested in checkboard matrix. The confluent cells were infected with the SARS-CoV-2 Wuhan strain in the presence of combination of the extract samples and the drug. After 48 h incubation, the culture fluids (ICF) were harvested and subjected to a plaque assay for virus quantification.

### 2.11. Statistical Analysis

All data were analyzed with GraphPad Prism (Version 10.5.0, La Jolla, CA, USA). Data were presented as mean values ± standard deviation. Statistical differences were evaluated by Student’s *t*-test. For all calculations, a two-tailed *p*-value less than 0.05 was considered statistically significant.

## 3. Results

### 3.1. Vitex-Hexane, Vitex-DCM, and Macaranga Extracts Inhibit Replication of the SARS-CoV-2 Wuhan Strain at Non-Toxic Concentrations

**Figure 1:**
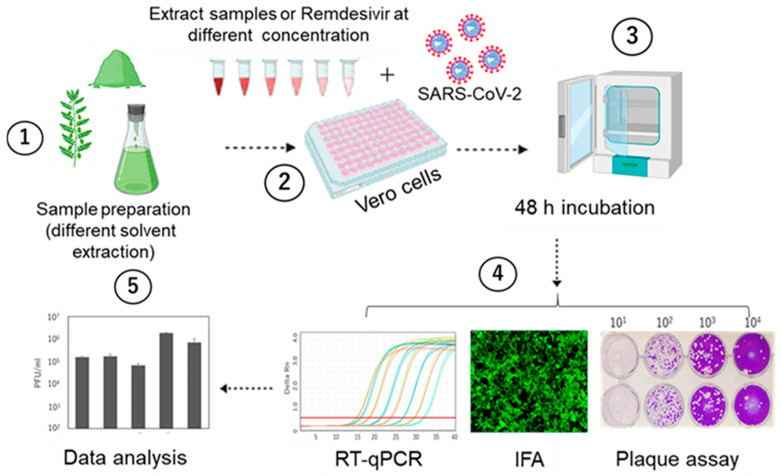
Schematic representation of the workflow for in vitro drug testing of plant extracts against SARS-CoV-2 using the Vero-E6 cell model: (1) plant extracts were prepared using different solvents, (2) confluent Vero-E6 cells were infected with SARS-CoV-2 in the presence of the samples, (3) the cells were incubated for 48 h, (4) viral inhibition was measured using various methods, including plaque assay, RT-qPCR, and immunofluorescence assay (IFA), and (5) data were analyzed and interpreted.

In extracting phytochemicals from plants, hexane is generally used to extract nonpolar compounds such as lipids, fatty acids, waxes, sterols, and carotenoids [28,29]. Dichloromethane, on the other hand, is effective in extracting compounds of moderate polarity such as alkaloids, flavonoids, phenolic acids, and coumarins [30,31]. In a preliminary study, we tested seven extracts prepared using different solvents from Philippine medicinal plants using real-time quantitative polymerase chain reaction (RT-qPCR) methods. Among them, three extract samples, namely Vitex-Hexane, Vitex-DCM, and Macaranga, demonstrated activity against the SARS-CoV-2 Wuhan strain. Here, we further evaluated these three extracts using plaque assays and immunofluorescence assays to confirm their inhibitory activity including against different variants of SARS-CoV-2. A schematic representation of the experimental workflow is depicted in Figure 1.

**Figure 2:**
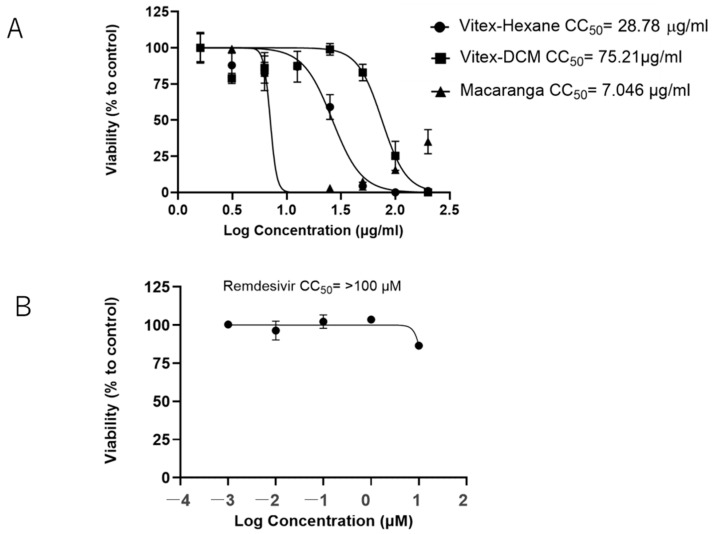
Cell viability assay. Vero cells E6 in 96-well plates were treated with Extracts-Hexane (EH), Extract-DCM, or Extract-M (**A**) and remdesivir (**B**) with increasing concentration. After 48 h incubation, cell viability was determined by MTT assay as described in the “Materials and Methods” section. The results are presented as the mean (±SD) of two independent experiments.

The Vitex-Hexane exhibited minimal cytotoxicity at a concentration of 12.5 μg/mL with a CC_50_ (50% cytotoxic concentration) of 28.78 μg/mL (Figure 2A). Vitex-DCM showed no significant cytotoxicity up to a concentration of 50 μg/mL (CC_50_ = 71.21 μg/mL). In contrast, Macaranga showed higher toxicity, with a CC_50_ of 7.05 μg/mL. Remdesivir, used as positive control for virus inhibition in this study, did not affect cell viability at the evaluated concentration (CC_50_ > 10 μM) (Figure 2B).

**Figure 3:**
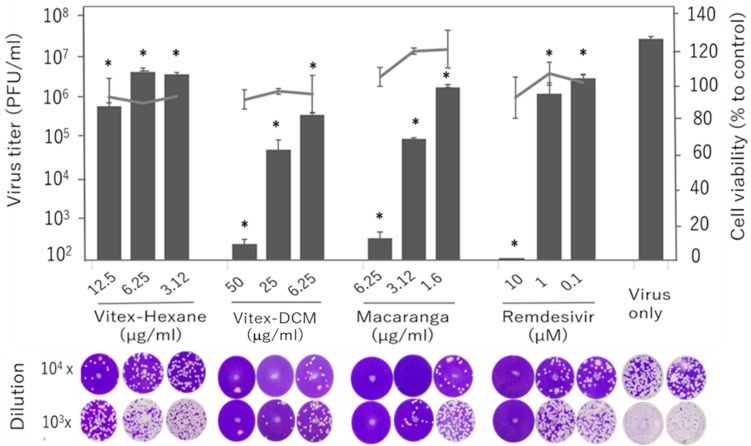
Evaluation of the antiviral activity in the (1) Vitex-Hexane extracts, (2) Vitex-DCM extracts, and (3) Macaranga extracts against SARS-CoV-2, assessed by using standard plaque assays and Vero E6 cells. Cells were treated with extracts at their maximum non-toxic concentrations. Remdesivir was used as a positive control. After 48 h, the level of infectious virus in the culture fluids was determined. The upper panel shows viral titers and cell viability; the lower panel shows representative plaques. Data are mean ± SD from two independent experiments. *: A *p*-value of <0.05 vs. the control is considered statistically significant.

We tested the antiviral activity of the three extracts against the SARS-CoV-2 Wuhan strain at three different concentrations, starting from the maximum non-toxic concentration for each extract: 12.5 µg/mL for Vitex-Hexane, 50 µg/mL for Vitex-DCM, and 6.25 µg/mL for Macaranga. Compared to the untreated infected control, Vitex-DCM and Macaranga demonstrated a 5-log reduction in viral load at the highest concentrations tested, as measured by plaque assay. Meanwhile, Vitex-Hexane exhibited moderate antiviral activity, with a 2-log reduction at a concentration of 50 µg/mL. Remdesivir showed nearly 100% inhibition at a concentration of 10 µM. Virus inhibition by all samples was shown in a dose-dependent manner (Figure 3).

**Figure 4:**
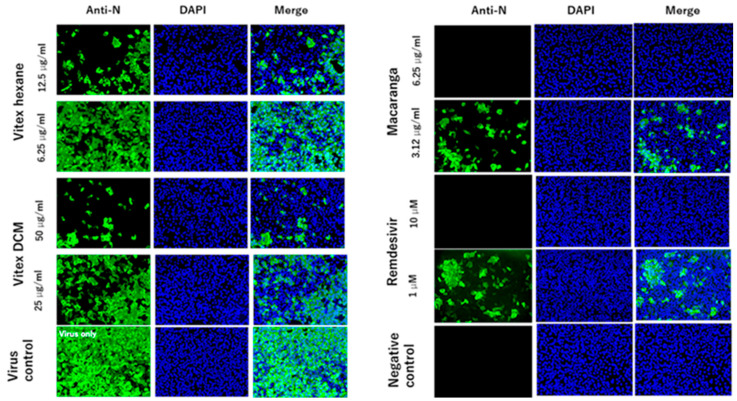
Antiviral activity of Extract-Hexane, Extract-DCM, and Extract-M against SARS-CoV-2 was evaluated using an immunofluorescence assay. Vero cells were infected with the Wuhan strain (MOI 0.05) and treated with extracts at the indicated concentrations. Remdesivir served as a positive control. After 48 h, cells were stained with anti-nucleocapsid antibody (green) and DAPI (blue) for fluorescence microscopy.

To confirm the antiviral activity of the three extracts, IFA assays were performed. In line with the virus titration results, IFA staining demonstrated a significant reduction in viral protein levels with the addition of the extracts at the indicated concentrations, as shown by the green fluorescence. No cells appeared to express the protein when infected with the virus in the presence of 6.25 μg/mL of Macaranga or 10 μM of remdesivir (Figure 4).

### 3.2. Inhibitory Effects of Vitex-Hexane, Vitex-DCM, and Macaranga Extracts Against SARS-CoV-2 Variants

**Figure 5:**
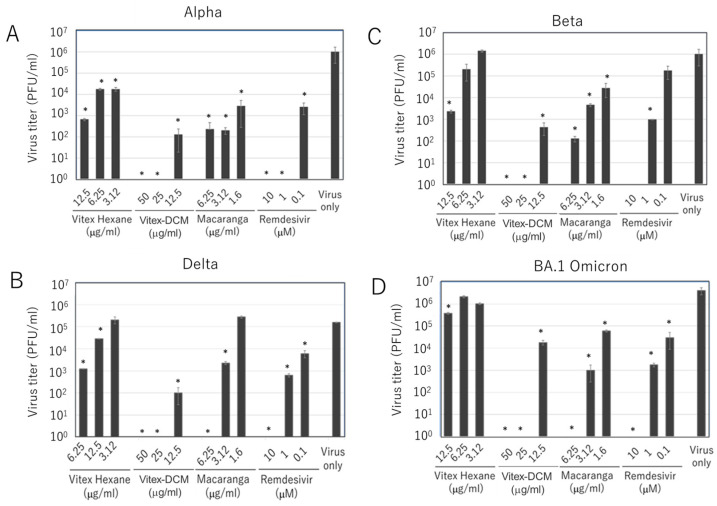
Antiviral activity of the extracts against SARS-CoV-2 variants. Vero E6 cells were infected (MOI 0.05) with Alpha (**A**), Beta (**B**), Delta (**C**), or Omicron BA.1 (**D**) variants and treated with the extracts at indicated concentrations. Remdesivir was used as a control. After 48 h, the infectious virus in the culture fluids was measured by plaque assays. Data are shown as mean ± SD of two independent experiments virus titers in the infectious culture fluid were measured by plaque assay. Data are shown as mean ± SD of two independent experiments. *: A *p*-value of <0.05 vs. the control is considered statistically significant.

We next evaluated the antiviral activity of Vitex-Hexane, Vitex-DCM, and Macaranga against SARS-CoV-2 variants, with remdesivir again used as a viral inhibition control. For this purpose, four variants—Alpha, Beta, Delta, and Omicron BA.1—were tested. As shown in Figure 5, all three extracts inhibited the replication of these variants in a dose-dependent manner, as determined by plaque assay, although the degree of inhibition varied among the variants. Taken together, the results clearly demonstrate that Vitex-Hexane, Vitex-DCM, and Macaranga inhibited SARS-CoV-2 infection, including both the original Wuhan strain and its variants, as confirmed by various methods.

### 3.3. Pre- and Post-Entry Inhibitory Effects of the Extracts

**Figure 6:**
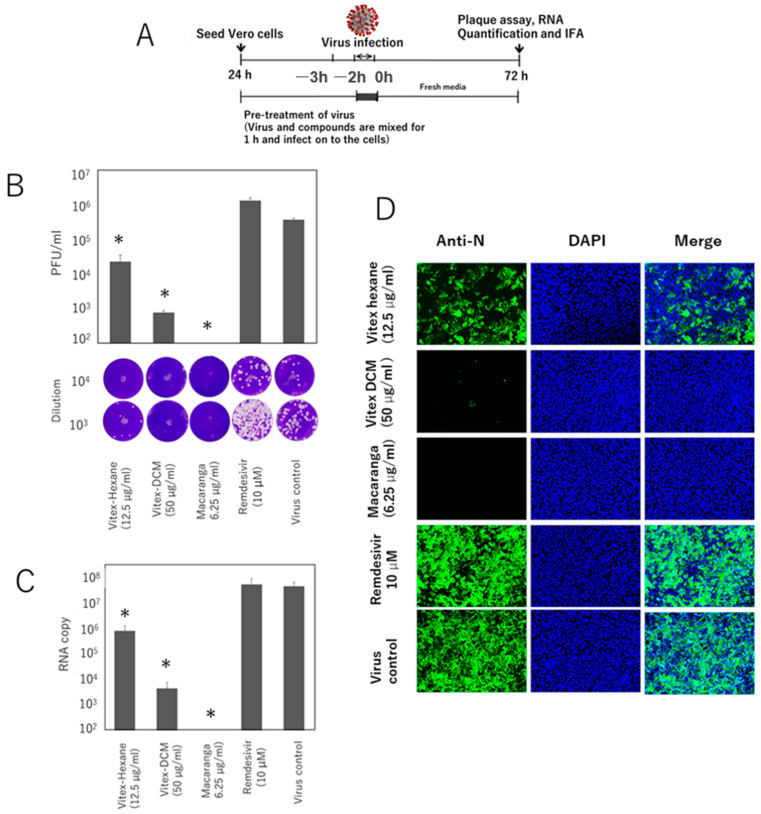
Time-of-addition assay (virus pre-treatment). (**A**) Schematic diagram of the viral inhibition assay. SARS-CoV-2 (MOI 0.05) was mixed with extracts or remdesivir at the indicated concentrations for 1 h and added to Vero E6 cells. After 48 h inoculation, viral inhibition was assessed using (**B**) plaque assay showing viral titers (top) and representative plaques (bottom), (**C**) viral RNA levels by RT-qPCR, and (**D**) immunofluorescence staining targeting the nucleocapsid protein. Untreated infected cells served as infection controls. Data are shown as mean ± SD from two independent experiments. *: A *p*-value of <0.05 vs. the control is considered statistically significant.

**Figure 7:**
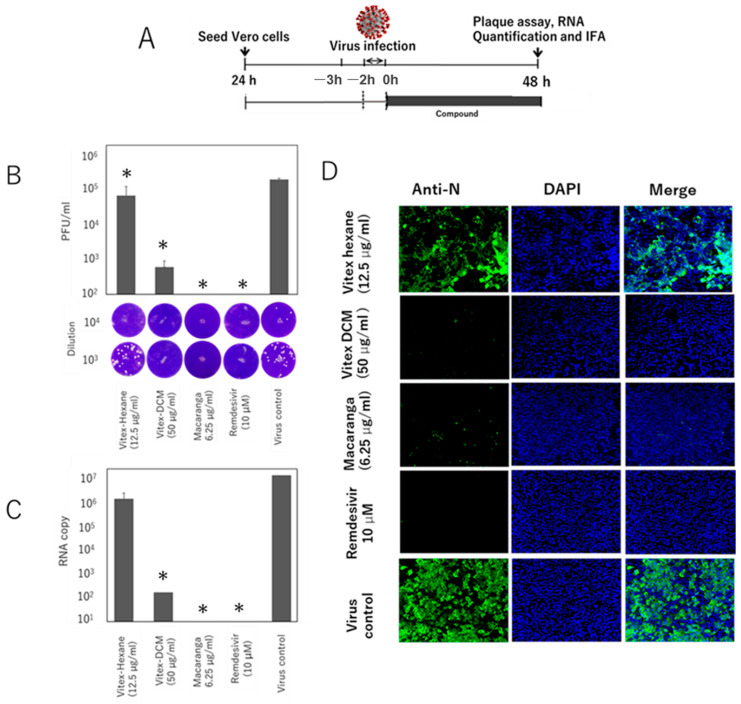
Time-of-addition assay (post-entry treatment). Vero E6 cells were infected with SARS-CoV-2 (MOI 0.05) for 1 h. After virus adsorption, the inoculum was removed, cells were washed, and fresh medium containing extracts or remdesivir was added. After 48 h, viral inhibition was evaluated. (**A**) Schematic of the post-entry treatment setup, (**B**) plaque assay showing viral titers (top) and representative plaques (bottom), (**C**) RT-qPCR for viral RNA levels, and (**D**) immunofluorescence staining for nucleocapsid protein. Data are shown as mean ± SD from two independent experiments. *: A *p*-value of <0.05 vs. the control is considered statistically significant.

To investigate the stage of the SARS-CoV-2 replication cycle at which the extracts exert their antiviral effects, time-of-addition studies were conducted using different treatment strategies at the maximum non-toxic concentrations of each extract (Figure 6A and Figure 7A). Viral inhibition was evaluated using plaque assay, RT-qPCR, and IFA. A significant reduction in viral growth was observed when the virus was pre-incubated with the extracts prior to infection. As determined by plaque assay, Vitex-Hexane and Vitex-DCM reduced the virus titer by approximately 1 log and 2.5 logs, respectively, as compared to virus infection control. Meanwhile, Macaranga completely inhibited viral growth. No inhibition was shown by remdesivir in this pretreatment study (Figure 6B). Consistently, the same inhibitory effects were confirmed by RT-qPCR for extracellular viral RNA quantification (Figure 6C) and IFA staining to detect viral protein expression in infected and treated cells (Figure 6D).

As shown in Figure 7B, a similar inhibitory effect was also observed in the post-entry treatment study. As determined by plague assay, treatment of the cells after virus infection resulted in a significant reduction in virus growth, particularly for Vitex-DCM, with a 2.5-log reduction in virus titer when compared to the infected-cell control, and for Macaranga, which completely inhibited virus growth. In contrast, Vitex-Hexane did not show significant inhibition. Additionally, remdesivir demonstrated complete inhibition in this post-entry treatment. The viral inhibition was further confirmed by RT-qPCR for extracellular viral RNA quantification (Figure 7C) and IFA staining (Figure 7D), both of which showed results consistent with the plaque assay.

### 3.4. Anti-SARS-CoV-2 RdRp Activity of Vitex-DCM and Macaranga

**Figure 8:**
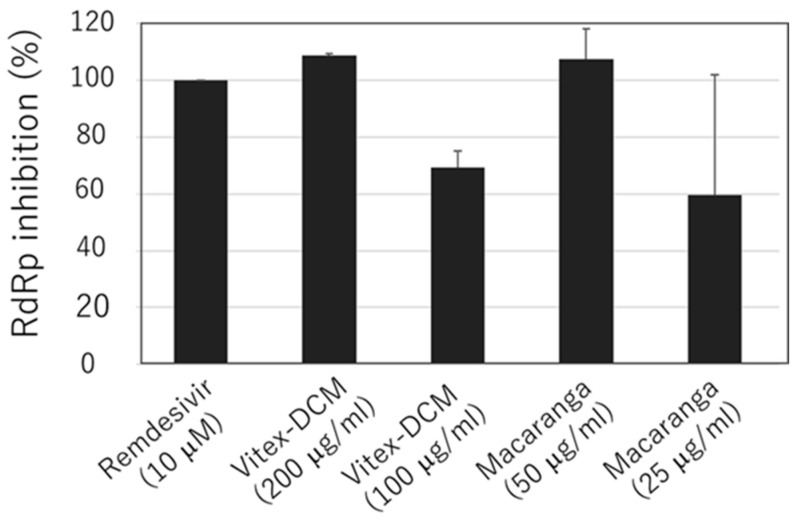
Inhibition of SARS-CoV-2 RNA-dependent RNA polymerase (RdRp) by Vitex-DCM and Macaranga. The inhibitory effects of Vitex-DCM at concentration of 200 μg/mL and 100 μg/mL and Macaranga at concentration of 50 μg/mL and 25 μg/mL were examined using an in vitro enzyme-based RdRp reporter assay. Purified RdRp complexes were incubated with varying concentration of the sample extracts and the resulting polymerization activity was quantified using fluorescent plate reader. The transcriptional activity of control samples treated with 0.1% DMSO was considered 0% for calculating relative enzymatic inhibition, while 100% inhibition was determined using positive control (10 µM Remdesivir). The results shown are the mean (±SD) of two independent experiments.

Since Vitex-DCM and Macaranga also demonstrated significant antiviral activity at the post-entry stage, in addition to their virucidal activity, we further investigated the mechanism by which viral inhibition occurs at this stage. Initially, we assessed the inhibition of SARS-CoV-2 3CL protease activity by Vitex-DCM and Macaranga, but no inhibitory activity was observed. We then evaluated their potential activity to inhibit the SARS-CoV-2 RdRp enzyme. Vitex-DCM inhibited SARS-CoV-2 RdRp activity by 100% at 200 μg/mL and by 70% at 100 μg/mL, while Macaranga reduced the enzyme activity by 100% at 50 μg/mL and by 60% at 25 μg/mL (Figure 8). This indicates that RdRp inhibition may contribute to viral suppression at the post-entry stage.

### 3.5. Synergistic Effect of Vitex-DCM and Macaranga with Remdesivir

**Figure 9:**
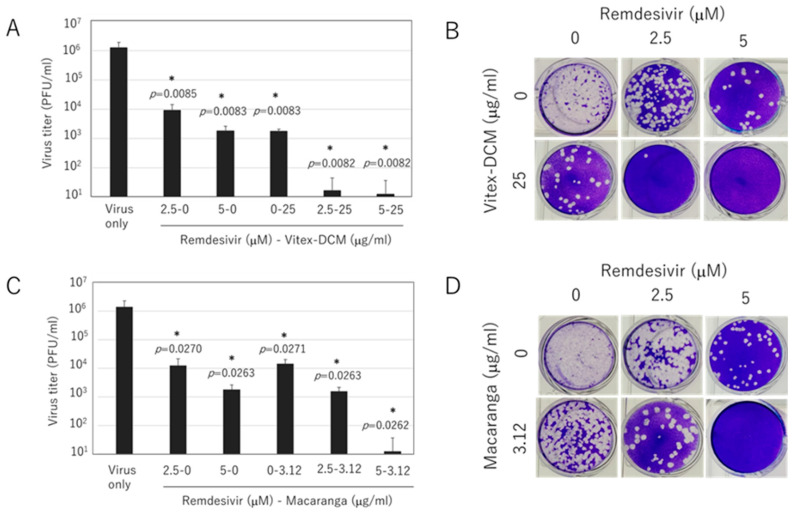
Synergistic effect of Vitex-DCM or Macaranga with remdesivir. Vero E6 cells were exposed to the SARS-CoV-2 Wuhan strain at a multiplicity of infection (MOI) of 0.05 in the presence of a combination of extract samples and remdesivir at the indicated concentrations. (**A**) Viral titers measured by plaque assay on Vero-TM cells and (**B**) representative plaques for the combination of Vitex-DCM and remdesivir. (**C**) Viral titers by plaque assay and (**D**) representative plaques of the combination of Macaranga and remdesivir. The results are presented as the mean (±SD) of two independent experiments. *: A *p*-value of <0.05 vs. the control is considered statistically significant.

Finally, the potential of Vitex-DCM and Macaranga working in synergy with remdesivir to inhibit the replication of SARS-CoV-2 was explored. For this purpose, we conducted a drug–drug interaction test through a combination assay and measured viral titer by plague assay. As shown in Figure 9A,B, the combination of Vitex-DCM at concentration of 25 µg/mL with remdesivir (2.5 and 5 µM) exhibited significantly enhanced inhibition compared to those treated with a single drug or sample. A similar synergistic effect was also observed with Macaranga. The combination of Macaranga (3.12 µg/mL) with remdesivir (2.5 and 5 µM) enhanced inhibition, with a more pronounced effect at 5 µM of remdesivir, compared to treatment with either agent alone (Figure 9C,D). The combination of remdesivir with DCM or Macaranga at the tested concentrations did not exhibit toxicity. These results suggest the potential use of Vitex-DCM and Macaranga in combination with remdesivir to enhance drug efficacy without affecting cytotoxicity.

## 4. Discussion

For centuries, long before the COVID-19 pandemic, natural products and herbal extracts have been widely used as traditional and complementary medicines in Asian and African countries, particularly for treating a variety of diseases caused by infectious agents [32,33]. For instance, *Artemisia annua* L. has a rich ethnobotanical history and has been traditionally used to treat fevers associated with infectious diseases such as malaria [34]. More recently, it has been shown to possess antiviral properties against SARS-CoV-2 [35]. The emergence of COVID-19 caused by the new coronavirus underscores the need to consider plants as potential sources for drug development, given their proven history as sources of effective pharmaceuticals.

As an RNA virus, SARS-CoV-2 is prone to genetic evolution through mutation over time while adapting to its new human hosts. This has resulted in the emergence of several variants, including Alpha, Beta, Gamma, Delta, and Omicron, which have been associated with enhanced transmissibility and increased virulence [36], evasion of neutralization antibodies [37], emergence of escape mutations during monoclonal antibody monotherapy [38], and in vitro and clinical antiviral resistance [39,40,41]. Therefore, a broader diversification of therapeutics is needed.

In the present study, three selected plant extracts prepared with different solvents showed inhibition against SARS-CoV-2 Wuhan strain, with Vitex-DCM and Macaranga demonstrating relatively stronger activity, as determined by various methods, including plaque assay, immunofluorescence assay, and RT-qPCR. This is further validated by the antiviral activity against four major variants—Alpha, Beta, Delta, and Omicron BA.1—suggesting that Vitex-DCM, Macaranga, and Vitex-Hexane could be considered as sources of lead candidates for the development of an anti-SARS-CoV-2 drug. A previous study has reported that the chloroform extract of Vitex negundo leaves exhibits antiviral properties against the dengue virus [42]. Our study is the first to demonstrate that extracts from Vitex negundo possess in vitro antiviral activity against SARS-CoV-2. Furthermore, this is also the first study to report the antiviral property of Macaranga extract. Therefore, its potential antiviral activity against other viruses warrants further exploration.

The SARS-CoV-2 replication cycle begin with the attachment of the receptor-binding domain (RBD) of the viral Spike (S) protein to the host membrane receptor, angiotensin-converting enzyme 2 (ACE2). Host proteases then cleave the Spike protein, allowing the virus to fuse with the host cell membrane and release its genome into the cytoplasm. Inside the host cell, the viral RNA is translated into polyproteins, which are cleaved into individual non-structural proteins (nsp 1–16) by viral proteases (PLpro and Mpro). These proteins form the replicase–transcriptase complex (RTC), which replicates the viral genome and produces sub-genomic RNAs. The structural proteins (Spike, Envelope, and Membrane) are produced, inserted into the endoplasmic reticulum (ER), and transported to the ER-Golgi intermediate compartment (ERGIC), where they combine with the replicated RNA to form new virus. The mature viruses are then packaged into vesicles and released from the cell by exocytosis [43,44]. Each stage of this process can potentially be targeted for inhibition.

To determine which step of the viral replication cycle is inhibited by our extract samples, we conducted time-of-addition studies. The results showed Vitex-DCM and Macaranga exerted its antiviral activity both during the pre-infection (when preincubated with the virus prior to infection or co-treatment) and post-infection stage, while Vitex-Hexane primarily acted at the pre-infection stage. No substantial inhibitory effect was observed when the extracts were applied to the cells prior to infection. The inhibitory effect of Vitex-DCM and Macaranga at both the pre- and post-infection stages suggests that these extracts inhibit SARS-CoV-2 through multiple mechanisms.

The significant reduction in infectivity observed during pre-treatment indicates strong virucidal activity by Vitex-DCM and Macaranga against SARS-CoV-2. One possible explanation is that the components of the extracts may directly interact with structural glycoproteins or other viral envelope components, disrupting the interaction between the virus and host cells and thereby impairing infection. This mode of action has been commonly reported for plant-derived compounds against various viruses [45]. Such a mode of antiviral action can be attributed to the bioactive substances produced by medicinal plants. In addition to producing primary metabolites essential for basic physiological functions, plants also generate secondary metabolites—non-essential compounds that are not crucial for plant survival but often demonstrate significant bioactivity. These secondary metabolites include molecules such as saponins, coumarins, flavonoids, alkaloids, steroids, antibiotics, resins, essential oils, tannins, minerals, and vitamins [46]. Secondary metabolites such as alkaloids and flavonoids have been identified to possess virucidal properties [47].

In addition to their virucidal activity, Vitex-DCM and Macaranga strongly inhibited the virus at the post-entry stage, suggesting they may interfere with the viral replication cycle and/or assembly. The active components in the extracts may interact with viral or host proteins involved in these processes, thereby inhibiting virus replication. Although no inhibitory activity was observed for SARS-CoV-2 3CL protease, Vitex-DCM and Macaranga inhibited the transcriptional activity of purified SARS-CoV-2 RdRp, which consists of the NSP12 catalytic subunit and two additional proteins, NSP7 and NSP8. The results indicate that the active compound(s) of Vitex-DCM and Macaranga may bind to or interact with the SARS-CoV-2 RdRp protein complex and inhibit its enzymatic function. Natural isolate compounds, such as polyphenols, alkaloids, and indoles, have shown potential as anti-SARS-CoV-2 RdRp inhibitors in both in silico and in vitro studies [48]. For example, an in vitro study reported that baicalein and baicalin, two polyphenolic flavonoids extracted from Scutellaria baicalensis root, inhibited SARS-CoV-2 RdRp activity [49]. Since RdRp is a key enzyme in viral replication, our findings, along with previous studies, highlight the strong potential of plant-derived compounds as promising drug candidates for combating SARS-CoV-2 by targeting its replication enzyme. However, further elucidation of the potential mode of action of our extracts still requires additional investigation. This will also require identifying the specific active fractions or substances from our extract samples.

The combined use of antiviral compounds with distinct mechanisms of action may work synergistically, offering benefits beyond those of single-agent treatment. It has been proven that drug combinations are highly effective as antiviral therapeutics for hepatitis C infections and human immunodeficiency virus (HIV) [50], as well as SARS-CoV-2 [51,52,53]. Our findings illustrate the synergistic effect of the extracts (Vitex-DCM and Macaranga) when administered as a co-treatment with remdesivir, an adenosine analogue approved for the treatment of COVID-19. Remdesivir has demonstrated clinical efficacy in reducing the risk of progression to severe illness and hospitalization in high-risk individuals [54,55]. A drug-combination approach for remdesivir is therefore promising for disease treatment. In addition to increasing drug efficacy, combination therapy could be a key strategy to reduce the emergence of resistant viral mutants. It is widely recognized that the constant development of drug resistance is highly likely when single-molecule therapeutic agents are used. Since a single molecule targets only one site, the resulting drug exerts strong evolutionary pressure on the virus to develop resistance. Therefore, the multi-target therapy holds promise for the prevention of antiviral resistance. In recent years, this strategy has gained increasing acceptance as a viable approach for antiviral treatment. For example, a combination of ganciclovir and trifluridine had been shown be an effective strategy to prevent the development of drug resistance in herpes simplex virus 1 (HSV-1) infection [56].

In this study, we used Vero E6 cells as a platform for antiviral screening against SARS-CoV-2, with remdesivir as a positive control. Our cell culture model showed that remdesivir exhibited minimal toxicity at concentrations up to 10 µM, consistent with previous studies that used the same Vero E6 cell line and virus strain [57]. At this concentration, our results demonstrated nearly 100% inhibition of the Wuhan strain as well as all tested variants. In addition, our time-of-addition assay showed that remdesivir functioned at a post-entry stage but not during pretreatment, consistent with its inhibitory activity against SARS-CoV-2 RdRp as a nucleotide analogue, which incorporates into nascent viral RNA chains leading to premature viral RNA synthesis [58]. Collectively, these results confirm that our in vitro antiviral testing system is reliable and facilitates mode-of-action studies.

## 5. Conclusions

This study demonstrates the potential inhibitory effects of Vitex-Hexane, Vitex-DCM, and Macaranga against SARS-CoV-2 through different treatment strategies, suggesting inhibition at various stages of the viral replication cycle. These effects appear to be independent of viral variants. The synergistic interaction of Vitex-Hexane or Macaranga with remdesivir highlights the clinical potential of drug combination therapy in combating SARS-CoV-2 and preventing the emergence of drug resistance. Further studies are needed to evaluate their efficacy in vivo and identify their bioactive compounds.

## Data Availability

The datasets generated and/or analyzed during the current study are available in the manuscript.
